# Marketing claims, promotional strategies, and product information on Malaysian e-cigarette retailer websites-a content analysis

**DOI:** 10.1186/s13011-024-00592-z

**Published:** 2024-01-25

**Authors:** Sameeha Misriya Shroff, Chandrashekhar T Sreeramareddy

**Affiliations:** 1 School of Postgraduate Studies, International Medical University, Jalan Jalil Perkasa 19, Kuala Lumpur, 57000, Malaysia; 2 Division of Community Medicine and Public Health, International Medical University, Jalan Jalil Perkasa 19, Kuala Lumpur 57000, Malaysia

**Keywords:** E-Cigarette use, Online Marketing, Online sales, Regulation, Vape.

## Abstract

**Background:**

Marketing and sales of e-cigarettes are unregulated in Malaysia. We analyzed content displayed on e-cigarette retailer websites to identify marketing claims, promotional strategies, and product details in the year 2022.

**Methods:**

We analyzed 30 Malaysia-based retailer websites using a mixed methods approach. Data were extracted as the frequency of occurrences of marketing claims, presence of regulatory information, product types, and flavors of e-juice as per a predefined codebook based on published literature. We also extracted textual details published on the websites about marketing claims, and slogans.

**Results:**

Most retailer websites provided contact information and physical store addresses (83%) but only half had ‘click through’ age verification (57%) that seldom needed any identification proof for age (3%). Marketing claims were related to health (47%), smoking cessation (37%), and modernity/trend (37%) and none had health warnings. Promotional strategies were discounts (80%). starter kits (57%) and email subscriptions (53%). Product types displayed were rechargeable (97%) and disposable (87%) devices and e-liquids (90%) of an array of flavors (> 100). Nicotine presence, its concentration, and “nicotine is an addictive chemical” were displayed in 93%, 53%, and 23% of websites respectively.

**Conclusion:**

Surveillance of content displayed online on e-cigarette retailer websites and regulation of online marketing and sales should be implemented by the Ministry of Health, Malaysia. Such measures are needed to prevent access to, and initiation of e-cigarette use among the youth and adults who do not smoke.

**Supplementary Information:**

The online version contains supplementary material available at 10.1186/s13011-024-00592-z.

## Introduction

A gradual decline in cigarette smoking [1] has marked a rise in the use of electronic cigarettes (e-cigarettes) usually among the youth in high-income countries (HICs) [2]. In 2020 it was estimated that globally there were 68 million e-cigarette users [3]. The global prevalence of the past 30 days use of e-cigarettes was 9.8% among youth aged 12–16 years [4, 5]. The youth who are exposed to e-cigarette advertisements are more likely to experiment with or use e-cigarettes in the future [6]. Claimed as a safer alternative or harm reduction or smoking cessation tool for addicted conventional cigarette smokers [7] e-cigarette marketing and sales are not usually strictly regulated worldwide [8]. The internet has been the main challenge for the implementation of regulations on marketing and sales of e-cigarettes as the manufacturers usually circumvent the regulatory policies [9]. In HICs, marketing of e-cigarettes widely prevails on social media platforms, and it is linked to youth e-cigarette uptake [10]. Social media marketing is also reported to be present in middle-income countries [11].

The use of e-cigarettes may assist in harm reduction and smoking cessation among cigarette smokers, but emerging evidence suggests that potential risks are higher than the benefits for non-smokers [12]. The perceptions of non-smokers about e-cigarette uptake are often influenced by the messages displayed about e-cigarettes by the manufacturer or retailer websites [13]. Therefore, it is important to examine the claims or propositions either implied or overt [14–17] made by the retailers. E-cigarette manufacturers have also been very versatile by introducing designs and flavors of e-cigarettes to improve their attractiveness, appeal, and uptake [18]. Previous research on online media has reported such information is relevant to local as well as global contexts [14–17]. Studies have reported the extent of compliance with the existing regulations for marketing and sales on online media [19, 20]. There are a few reports on product types in China, Indonesia, and Nigeria [16, 21, 22] and selling propositions in South Africa [15]. Another study has reported on social media marketing in India, Indonesia, and Mexico [11].

Malaysia has a big e-cigarette market with a prevalence of daily use at 5.8% in 2020 among adults and past 30-day e-cigarette use at 9.1% among youth aged 13–19 years [23]. E-cigarette marketing and sales are not regulated in Malaysia. In 2022, the Malaysian government proposed a ban on cigarette smoking including e-cigarettes in its proposed ‘tobacco end game’ strategy [24]. However, the bill is yet to be passed. In a regulatory void, very little is known about marketing propositions, product types and, flavors. Therefore, it is important to study the content of e-cigarette manufacturer websites to inform policymakers about regulations for internet-based e-cigarette retailers. Public health efforts for targeted prevention and mitigating the prevalence of e-cigarette use are not realizable without reporting the e-cigarette-related information, advertising, and products presented on online media. We aimed to determine the marketing claims, promotional strategies, and product details displayed on online retailer websites originating from Malaysia in the year 2022.

## Methods

### Study design and sample selection

We did content analyses of information displayed on e-cigarette retailer websites to identify qualitative and quantitative information about e-cigarettes. During October and November 2022, we identified the e-cigarette manufacturers and retailer websites on the Google search engine (https://www.google.com.my/?hl=ms). We used the search terms ‘vape shop Malaysia’, ‘e-cigarette shop Malaysia’, ‘Malaysia vapes online’, ‘Malaysia e-cigarette online’, ‘vape Malaysia’, ‘e-cigarette Malaysia’, ‘e-juice Malaysia’. The first 15 pages of Google search results for every keyword search were manually screened to be included in content analyses. Beyond the first 15 pages, the sites were irrelevant and duplicates. The websites included were based on the criteria that they are either e-cigarette manufacturers or retailer websites whose web addresses are based in Malaysia and exclusively sell e-cigarette-related products directly to customers in Malaysia. Websites in both Bahasa Malaysia national language and English language were included. A total of 110 results were yielded per keyword search resulting in a total of 1,100 search result hits. Applying the inclusion criteria and removing duplicates and overlapping results, a total of 30 websites were identified. Each page of the included websites was archived using the web clipping software Microsoft OneNote for detailed review for content analyses using the codebook.

### Coding instrument

The codebook about marketing claims, and promotional strategies was adapted from similar studies from the United States [14] and China [16]. This codebook was adapted to be consistent with previous literature and ensure comparability. Using the codebook we identified thematic content, claims, and other descriptors such as contact information, product types, their cost and instructions, flavor information, age verifications, nicotine content, disclaimers, and any other information of interest that was displayed on the included websites. Regarding the marketing claims themes such as ‘e-cigarettes are healthier’ and ‘lesser toxic element’ were coded the health-related claims. Themes such as ‘e-cigarettes can assist in reduction of cigarette smoking’ and ‘help to quit smoking’ were coded as cessation-related. The operational definitions and example text for each code are provided in Table 1.

**Table 1:** The definitions of themes, regulatory language, product-related information/content, and marketing-related slogans, strategies, and claims with text examples (if appropriate)

Theme	Definition	Sub-themes	Definition
Age restriction*	Age verification that restricts minors from accessing the website	Click through verification ID requirement	Confirmation of age by click through option “are you above 18 years old? Yes/ No.” ID such as IC, driver’s license, student ID or other proof of age.
Contact Information*	Display of contact information for customers to directly contact e-cigarette vendor	Phone number, Email address, Physical store address	
Regulatory language	Statements of warnings or disclaimers related to minors, e-cigarette product and nicotine	Age warning, Nicotine Disclaimers, Health warnings, Noticeable regulatory language	Disclaimers against minors’ use of e-cigarettes. Disclaimers about the presence of nicotine in any of the products. Warning about the possible side effects of using e-cigarettes. Presence of warnings and disclaimers in large, visible text that catches the eye, and is on the homepage.
Claims	Any display of content with regards to the health effects, safety, and convenience of e-cigarette use, trendy, or modernity	Health related, Cessation related, Smoke anywhere, Reduced SHS, affordability, Convenient to use, Modern, Social enhancement	E-cigarettes are healthier than conventional cigarettes and contain fewer toxic chemicals. E-cigarettes can reduce cigarette smoking and aid in smoking cessation. E-cigarettes can circumvent clean air policies and can be used where conventional cigarette smoking is prohibited. reduced exposure to second hand-smoke and less dangerous vapors. E-cigarettes being cheaper than conventional cigarettes. E-cigarettes are easier to carry and dispose, and that they are more accessible to purchase. Messaging that using e-cigarettes is a modern, trendy, revolutionary way of smoking or a technologically advanced method of smoking. Claims that e-cigarette smoking can increase social status, make one popular or increase one’s sexual appeal.
Product types	Variety of products that are available for purchase on the website	Starter kit, Rechargeable e-cigarettes, Disposable e-cigarettes, E-liquid	Vape kit for beginners containing all the components required for first-time vaping. Vape devices that can be reused multiple times by recharging and require only one-time purchase. A non-rechargeable vaping device that is pre-filled with e-liquid and is designed for one-time use only.
Flavors	Variety of popular e-liquid flavors available for purchase on the website.	Fruit, Coffee, Menthol/ mint, Tobacco, Unflavored, and other flavors.	Flavored or unflavored liquid used to produce vapor in e-cigarettes also known as e-juice or vape juice (may contain nicotine.)
Ingredient disclosure*	List of ingredients/ chemical components of e-liquid displayed on the website for each product.		
Nicotine content*	Disclosure of nicotine presence and content in the products available for purchase.	Nicotine products, Nicotine content in milligrams, Nicotine use promotion.	
Instruction to use*	Display of detailed instruction of how to use vape products.		
Promotions and discounts	Promotional offers and discounts of products	Discounted price, Free samples, Social media, Blog, Newsletter subscriptions	
Links to the online community*	External links to online communities and platforms		Links to Facebook, Twitter, Instagram, and YouTube. A blog page for sharing discussions and information about e-cigarette and vaping-related topics. Timely subscriptions to newsletters that are emailed to consumers directly carrying information and updates about new products and offers.
Slogans*	Catchy slogans or phrases displayed on the website for advertising and appealing e-cigarettes to consumers		

*Themes originally developed for this study

### Coding procedure

The principal researcher (SMS) manually analyzed each page of the selected website saved into Microsoft OneNote. Using the code book, the presence/absence of themes and other relevant information and relevant textual content was extracted by the primary researcher (SMS). The data extracted was verified by the second researcher (CTS). Data on the themes were gathered in binary formats and textual information. In addition to the codes/themes, we gathered data on types of devices, prices, and celebrity endorsements if any displayed on the websites. The examples of identified themes saved as screenshots in which detailed information are displayed on all the included websites that were analysed are shown in Appendix A.

### Data analyses

Frequency and percentages of themes, claims related to regulatory (including contact details) and promotional strategies; types of products; prices; nicotine levels, and other contents were calculated on Microsoft excel. The textual content that was classified as regulatory language, slogans and promotional language was presented qualitatively.

## Results

### Contact and regulatory information

As shown in Table 2, the contact information such as electronic address and phone numbers were provided for the customer on 83% of the websites and physical store address was provided on 57% of the websites. Age verification to prevent minors from accessing the website was found in 60%, and age warning was provided in 43%. However, only one (3% ) website required identification proof to verify the age. Age verification was written as “*click Enter only if you are at least 18 years of age*”, whereas age warnings were displayed as a banner either on the top or bottom of the homepages of the websites with texts such as “*This product is not intended for minors*”. Alarmingly, health warnings were not present on any of the websites, and age verification and identification proof were not needed at all to access most websites.

**Table 2:** Descriptive statistics of regulatory language, product-related information/content, and marketing-related slogans, strategies, and claims on 30 websites

Regulatory and contact		Promotional claims		Promotional strategies		Product types and contents	
Themes	N (%)	Themes	N (%)		N (%)		N (%)
Age verification	18 (60)	Health-related claims	14 (47)	Starter kits	17 (57)	Rechargeable vapes	29 (97)
Identification proof	1 (3)	Smoking cessation claims	11 (37)	Discounts	24 (80)	Disposable vapes	26 (87)
Age warning	13 (43)	Reduced secondhand smoke	8 (27)	Free Samples	7 (23)	E-liquids	27 (90)
Health warning	0 (0)	Affordability	6 (20)	Social media links	26 (87)	Instructions to use	6 (20)
Physical address	17 (57)	Convenience to use	10 (33)	Blogs	7 (23)	Ingredient list	4 (13)
Email address	25 (83)	Modernity or trendy claims	11 (37)	Newsletter	16 (53)	Nicotine product disclosure	28 (93)
Phone number	25 (83)	Improved social status	4 (13)	Slogan/s	14 (47)	Nicotine content disclosure	16 (53)
						Nicotine promotion	11 (37)
						Nicotine disclaimer	7 (23)

### Claims about e-cigarettes

The frequencies of marketing claims that were coded are shown in Tables 2 and the example information supporting the codes that were presented on the websites are shown in Table 3. Most common claims made were related to health (47%), followed by smoking cessation (37%), and modernity or trendy (37%). The other claims made were about convenience to use (33%), reducing second-hand smoke (27%), affordability (20%), and improved social status (13%), (Table 2). Examples of statements such as “*according to Public Health England, vaping is 95% less harmful to the user than smoking*” and “*level of carcinogenic dust is 1000 times lower than in cigarettes*” were health-related claims. Statements such as “*our products have helped billions of smokers to quit smoking using e-cigarettes as TRT (Tobacco Replacement Therapy)*” and “*our e-cigarettes were able to help smokers to stop smoking in less than one month*” were smoking cessation-related claims. Eye catching statements such as “*Sleek design to elevate your vaping experience* " and “*Smartphone-level craftsmanship*” were used to make claims related to modernity (Table 3). Quotes such as “*Pocket-friendly pod system for vaping on the go*.” “*Lightweight and durable”*, portraying e-cigarettes as convenient to use particularly during travels. Quotes such as “*vaping can improve your image and make you more dateable*” and “*Vaping is a lifestyle*” were available to claim that e-cigarette use was a status symbol and of hedonistic value. The websites also contained quotes that portrayed e-cigarettes as environment friendly by citing that e-cigarette produces fewer chemicals, and is devoid of dust, or carbon monoxide, and thus less harmful to others. Websites also quoted that switching to e-cigarettes that are cheaper than conventional cigarettes could save money (Table 3).

**Table 3:** Textual content related to e-cigarette promotion claims and slogans identified on internet e-cigarette vendor websites

Promotional language	Text examples
Health-related claims	“According to Public Health England, vaping is 95% less harmful to the user than smoking.” “E-cigarettes could be a game changer in public health.” “Our products contain less harmful chemicals than cigarettes and are healthier.” “Level of carcinogenic dust is 1000 times lower than in cigarettes.” “Many benefits of vaping over smoking.” “Switch to e-cigarettes to reduce tobacco-related illnesses.”
Smoking cessation-related claims	“Our brand’s mission is to encourage smokers to stop smoking and start vaping.” “E-cigarettes solution to combustible smoking.” “5 ways to stop smoking slowly for Malaysians.” “Goal to create a world free of cigarettes by helping others stop smoking.” “Our products have helped billions of smokers to quit smoking using e-cigarettes as Tobacco Replacement Therapy.” “Our e-cigarettes were able to help smokers to stop smoking in less than one month.” “Start vaping to kick the cigarette habit.” “Our mission is to help you curb your smoking habits.”
Reduced SHS/ smoke-free claims	“According to Public Health England, vaping is less harmful to bystanders and produces fewer chemicals.” “Our company’s mission is to create a smoke-free ambiance.” “Reduced second-hand smoke compared to cigarette smoking creates a safer environment for those around.” “No carbon monoxide or chemical dust.”
Affordability claims	“Vaping vs smoking: which is more cost-effective?” “You will save ringgit in the long run.” “Switching to vape can help you save some ringgit which can be used for other activities like traveling to holiday destinations or enjoying good food with friends.” “Vaping is more economical than smoking cigarettes.” “E-cigarettes cheaper than tax-free, smuggled and premium cigarettes.”
Convenience of using claims	“Crafted for convenience.” “Pocket-friendly pod system for vaping on the go.” “Lightweight and durable for convenience during travels.” “Designed for society to enjoy the convenience brought by modern technology.” “Convenient and easy device to use.”
Modern/ trendy claims	“Modern alternative to the traditional cigarette.” “Smartphone level craftsmanship.” “Advanced technology of manufacture and engineering process from the lab to reality.” “Sleek design to elevate your vaping experience.” “Enhanced system for the better smoking experience.” “Wish to improve everyone’s life through modern and convenient electronic products.”
Social status claims	“Level up your dating game: Benefits of quitting smoking today and vaping.” “Vaping can improve your image and make you more dateable” “Vaping is a lifestyle.” “Growing number of millennials are hastening to make the switch to vape for FOMO (fear of missing out)” “Wake up your social life! Social advantages of making the switch to vape” “You might get a few compliments on the aroma”
Instructions to use the device	“How to use vape for the first time: Vaping dos and don’ts for beginners.” “How does a pod work instructional video.” “The best way to take a puff on our pods.” “Beginner’s guide for vaping: are you supposed to inhale when you vape?” “How do you make more vapor with e-cigarettes.” “How to use an e-cigarette to make it almost feel like smoking.” “What vape should I buy to quit smoking?”
Slogans	“Stop smoking & let’s change to vape.” “Future through the clouds.” “A better choice for smokers.” “Eat, Sleep, Vape, Repeat.” “Quit smoking. Let’s vape.” “Vape like royalty.” “Taste the real goodness.” “Say no to cigarettes. Vape is safer.”
Regulatory Language	
Age verification	“You must be 18 and above to view this website. Sale of products on this website is strictly prohibited to minors in compliance with local laws and regulations.” “This website contains adult material and is only suitable for those 18 years or older. Click Enter only if you are at least 18 years of age.”
Warning against minor use	“Age policy: This site contains information regarding electronic cigarettes. You must meet the minimum age requirement of your country and state to view any pages of the website and/or purchase electronic cigarettes and electronic cigarette-related products from this website.” “Vape not for MINOR!” “This product is not intended for minors.”
Ingredient disclosure	“Use of Federal Drug Administration-approved vegetable glycerin, propylene glycol, food-grade flavoring, and medical-grade nicotine salts or nicotine-containing pods. Menthol is also used for some flavors.”
Nicotine-related content	
Nicotine disclaimer	“Warning: This product contains nicotine. Nicotine is an addictive chemical.” Some products have nicotine warning labels on the product packaging.
Nicotine-related health claims	“Nicotine no more harmful than caffeine to health.” “Nicotine causes little to no harm, instead it is the smoke from cigarettes that is harmful.” “Nicotine in e-cigarettes less harmful than in tobacco.” “High-quality nicotine called PureNic is less harmful than traditional nicotine from tobacco.” “Guide to control nicotine intake with the help of e-cigarettes to overcome addiction.” “Promotional video on safety of PureNic compared to traditional tobacco.”
Nicotine promotion	“Faster absorption and stronger nicotine hit.” “Tastes and feels like a real cigarette.” “Nicotine head buzz you’ll get feels very similar to having your first puff of a cigarette in the morning.” “Use of nicotine science to provide maximum nicotine satisfaction, softer throat hit.” “Each pod contains nicotine equivalent to 40 cigarettes.” “Rechargeable device is equivalent to 550 cigarettes.” “Eco Nicotine aims in giving better taste and better satisfaction.”
Instructions for nicotine use	“Self-administered questionnaire to find out suitable nicotine content for vape depending on past cigarette smoking behavior.” “How do you know the nicotine content that you need?” “Instructions to choose the right level of nicotine.” “Should I choose Nicotine Salts or Freebase Nicotine?”
Flavors	energy drink, key lime pie, pink zest, root beet, strawberry cheesecake, “strawberry banana custard” “breakfast honey oat” “Aloe vera” “energy drink” “root beer” Mocha Praline, fruit custard, cereal milk, cookies cream, Raybina, American breakfast, Coco cola, green tea, milk pie, corn cheese, taro yam, soju, hagandaz, peanut butter, cacao, oatmeal, root beer, strawberry butter bread, Butterbeer, cheesecake, honey vanilla, Breakfast flavored (cinnamon glazed blueberry scone), dessert flavored (lemon tart, buttermilk pie, strawberry custard), Ribena, cola, Berry milk pie, blueberry milk pie, cannoli, lemon tart, cake pop, white gummi, punch bowl, butterscotch, cornbread, pina colada, Lassi juice, strawberry hazelnut chocolate, ice cream cereal, banana custard, lonjing tea, ice matcha, Red Energy, Apple zest, Custard, Pina colada, coconut, cream, custard.

### Promotional strategies

In addition to the positive claims made related to e-cigarettes most of the 30 websites studied used some form of promotional strategies shown in Table 2. The commonest was providing links to social media such as Instagram, Facebook, Twitter, and YouTube (87%) and discounts (80%). Other strategies were giving starter kits (57%), subscriptions by customer’s emails to receive updates and newsletters on new products and offers, (53%), and slogans and catchphrases (47%). “*Eat, Sleep, Vape, repeat*” and “*Quit smoking, Let’s Vaping*” were some examples of slogans exclusive to the two websites in addition to the other strategies employed by the 14 (47%) websites. Furthermore, free samples of e-cigarettes and liquids and blogs were also employed by about a quarter of the websites.

### Product types and nicotine-related information

The types of e-cigarette devices are shown in Table 2 while the range of flavors available are shown in Table 3. Most of the websites displayed rechargeable (97%), disposable (87%) types of devices, and e-liquids (90%). There was a very wide array of attractive flavors on offer for purchase, the commonest being Fruit (29), Coffee (24), Menthol/mint (26), Tobacco (21), and Candy (13). Instructions on how to use both nicotine and non-nicotine vape products were displayed on 6 (20%) and starter kits for beginners were sold on 17 websites (57%) (Table 2). Only four websites disclosed the information on the contents of e-juice as “propylene glycol”, and “vegetable glycerine”. One of the websites cited those soaps, sauces, salad dressing, etc. also contain these chemicals to claim the safety of e-cigarettes.

The nicotine-containing product was disclosed 93%.but only 7 (23%) provided a disclaimer that read a warning “This product contains nicotine. Nicotine is an addictive chemical”. On all these websites the disclaimer was placed at the top of the homepage. However, only 16 (53%) disclosed the actual nicotine content of varying strengths ranging from 3 to 50 milligrams per millilitre. The common strengths displayed were 20, 35, and 50 mg. Information related to the use of nicotine and messages that promoted the nicotine-containing products available for purchase on 11 (37%) websites (Table 2). These websites conveyed a range of messages regarding the nicotine present in e-cigarettes. Some interesting statements such as ‘*nicotine in e-cigarettes is less harmful than those in conventional cigarettes referring to a product known as “PureNic*” also included a video demonstrating its safety relative to nicotine in a conventional cigarette. Some statements related to faster absorption, greater ‘kick’, satisfaction, taste, etc. relative to conventional cigarettes regarding the nicotine contained in e-cigarettes (Table 3). In addition to the instructions on how to use e-cigarettes the websites also provided potential users options to choose from a range of products by type of device, price range, and the strengths of nicotine present in them. Interestingly one website provided a self-administered questionnaire to assist the consumers in deciding on nicotine content based on their past cigarette smoking behavior.

## Discussion

### Main findings

A content analysis of online marketing of e-cigarettes in Malaysia revealed that a wide range of e-cigarette products was offered to potential customers. Various promotional strategies, such as offering products in attractive flavors for youth appeal, promotional strategies via ‘starter packs’, discounts, and free gifts were employed; slogans, and catchphrases were also displayed to entice potential customers. Contact details and WhatsApp messenger links and physical addresses, links to social media, blogs, and email subscriptions were used as marketing strategies. Websites displayed positively framed claims related to health, smoking cessation, and hedonic values of e-cigarette; most websites disclosed that e-cigarette contains nicotine but not that nicotine is addictive.

### Health and smoking cessation-related marketing claims

Online marketing and sales of e-cigarettes are well-established strategies of tobacco companies [11, 25, 26]. Hence several researchers have studied online marketing, and content analyses of social media websites [25, 27]. To our knowledge, very little has been studied in detail about the content displayed on retailer websites in developing countries. Our analysis is the most up-to-date and comprehensive. Marketing claims reported by Grana et al. (2012) covered English websites based mainly in the United States and the United Kingdom [14], whereas Yao et al. (2013) covered Chinese websites [16] and Muposhi et al. (2017) studied South African websites [15]. In all these studies marketing claims made were like ours i.e., health benefits (*n* = 14), smoking cessation(*n* = 11), reduced exposure to secondhand smoke (*n* = 8), etc. All these claims appear to improve perceptions about perceived individual susceptibility to smoking-induced diseases and the benefits of quitting cigarette smoking according to the Health Belief Model. However, as in previous studies as well none of the websites presented any health effects of e-cigarettes as more evidence started to emerge [12]. Instead, e-cigarettes are being marketed favorably to entice customers with claims such as cleaner and healthier (no secondhand smoke) contrary to the evidence that e-cigarette aerosols are also a source of nicotine and other toxins including heavy metals [28]. Health benefits claimed by websites are substantiated by research evidence that e-cigarette assists cigarette smokers in reducing the number of cigarettes smoked per day and hence as a safer alternative for harm reduction [7]. E-cigarettes should not be promoted to non-smokers or those who have never used e-cigarettes considering short-term effects and the possibility of initiating cigarette smoking [29]. Smoking cessation claims made on the websites are not entirely incorrect but there is some evidence that nicotine-containing e-cigarettes offer benefits [29] to current cigarette smokers [7, 30]. Nevertheless, despite the evidence of short-term effects of e-cigarette use their long-term health impacts are not known. Celebrity endorsements were reported in previous studies [14, 16], the presence of doctors [14], and cartoon pictures [17]. However, retailer websites originating from Malaysia did not employ such tactics, but some claims were substantiated by citing Public Health England and the Journal of Nicotine and Tobacco Research.

### Marketing claims about lifestyle benefits, affordability, and convenience

Claims about the lifestyle benefits of e-cigarettes such as making claims of improved social status and modernity are reported in previous studies [11, 14–16]. In addition, the claims about affordability(*n* = 6), and convenience(*n* = 10), are aimed at encouraging the uptake of e-cigarettes by the non-smoking youth and young adults by making e-cigarettes appear healthier, less risky, and socially appealing. Celebrity endorsements were reported in previous studies [14, 16], the presence of doctors [14], and cartoon pictures [17]. However, retailer websites originating from Malaysia did not employ such tactics, but some claims were substantiated by citing Public Health England and the Journal of Nicotine and Tobacco Research.

### Age restrictions and promotional strategies

Websites mostly did not have strict checks to prevent access to minors as in previous studies about e-cigarette websites [17]. A review of published research has shown that most of the YouTube and Instagram have content that promotes e-cigarette use but seldom contain age and/or health warnings [25]. Currently, there is no law in Malaysia to prevent minors from purchasing e-cigarettes. Anecdotal evidence exists about the increasing number of minors using e-cigarettes in Malaysia [24]. Not placing age warnings and verification enables minors to procure e-cigarettes online [28]. Marketing strategies such as discounts (*n* = 20), free samples (*n* = 7), links to social media (*n* = 26), and myriad flavors(*n* > 100) were offered to make the e-cigarette more appealing to the youth. Together with claims about hedonistic claims, lack of age verifications, free samples, and starter kits act as a ‘bait to entice’ price-sensitive youth to initiate e-cigarettes. Most of the websites provide links to social media. The presence of e-cigarette marketing in social media has been reported in Indonesia, India, and Mexico as well [11].

### Nicotine content and disclaimers

Nicotine is a highly addictive substance [31], most websites disclosed that e-cigarette contains nicotine, but they seldom had disclaimers that ‘nicotine is addictive’ as in other studies [14, 17]. Rather websites describe various strengths and provide the buyers with options to customize the strength of nicotine content. Interestingly there were quotes implying the healthiness of nicotine present in e-cigarettes relative to conventional cigarettes and a positive impression about ‘nicotine to beat stress and anxiety’ as in studies from China and New Zealand [16, 18]. Such information is rather deceptive to naïve minors supporting the argument that the retailer websites were designed to promote online sales to younger individuals. The retailer websites contained information related to unconfirmed claims about the healthiness of e-cigarettes, and nicotine contained within them. Websites were promoting e-cigarettes via claims related to smoking cessation, reduced secondhand smoke as well as positive framing such as ease of use, convenience, affordability, and hedonic values.

### Policy implications

The lack of health warnings and age verifications, and the presence of a wide array of delivery systems, flavors, and links to social media confirms the design of retail websites to attract the youth and increase their appeal towards e-cigarettes. In the void of e-cigarette regulations in Malaysia, our findings provide important insights into the formulation of regulations about the information displayed and marketing strategies used on retailer websites. E-cigarette marketing online is not just limited to retailer websites as there are also physical stores where sales and marketing takes occurs [21, 32] and e-cigarette marketing is also prevalent in a variety of society media [11, 33] needs to be studied in Malaysia as well. Recently Malaysia has delisted gel and liquid nicotine from e-cigarettes from the ‘Poisons Act’ schedule that officially legalizes e-cigarettes that contain nicotine [34], while it has committed to the tobacco end game [35]. Nevertheless, strict control on information being displayed on retailer websites related to health and other benefits are to be regulated to avoid misinformation. Online marketing and sales regulations are needed to remove youth and non-smokers’ access to e-cigarettes. Restrictions are also needed on flavors, and disclosure that ‘nicotine is an addictive substance’.

### Limitations

The findings of the study are only limited to the retailers’ websites identified by our search. The results are not generalizable to the websites beyond those we analyzed as well as physical stores and social media. The tobacco industry’s marketing strategies are versatile to the regulatory environment. Hence, retailers’ website information reported in this paper may not be current. Finally, the coding applied was adapted from similar research in other countries and may not reflect all the possible information available on the websites. A standard codebook that can be adapted by the researchers to suit their local settings should be developed.

## Conclusion

The content displayed on the e-cigarette retailer websites needs to be under surveillance by the Ministry of Health, Malaysia. Relevant authorities should regulate the marketing and sales via online retail websites to prevent easy access and uptake of e-cigarettes by the youth and non-smoking adults.

### Electronic supplementary material

Below is the link to the electronic supplementary material.

Supplementary Material 1

## Data Availability

Data will be available from the authors upon request.

## Abbreviations

HICs: High-income Countries
TRT: Tobacco Replacement Therapy
