# Supplementary material for: Marketing claims, promotional strategies, and product information on Malaysian e-cigarette retailer websites-a content analysis
Source: Subst Abuse Treat Prev Policy. 2024 Jan 25;19:11. doi: 10.1186/s13011-024-00592-z (PMC10809498; doi:10.1186/s13011-024-00592-z)

Appendix to table 1.

1. **Age restriction:** The websites upheld age restriction by having click through age verification pop-ups prior to accessing the website. However, these verifications do not require any ID proof and simply consist of the user clicking that they are above 18 years old.

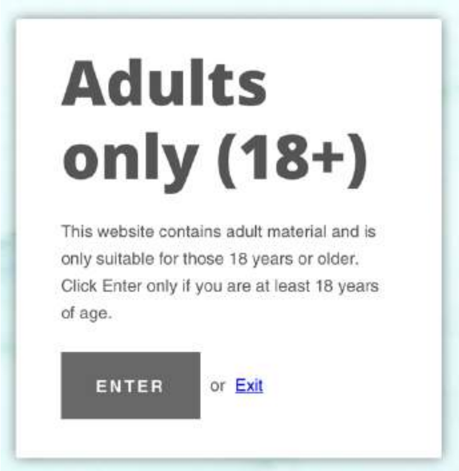

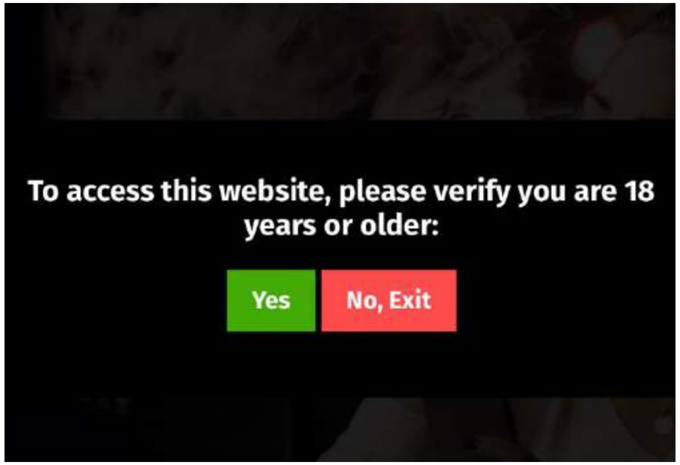

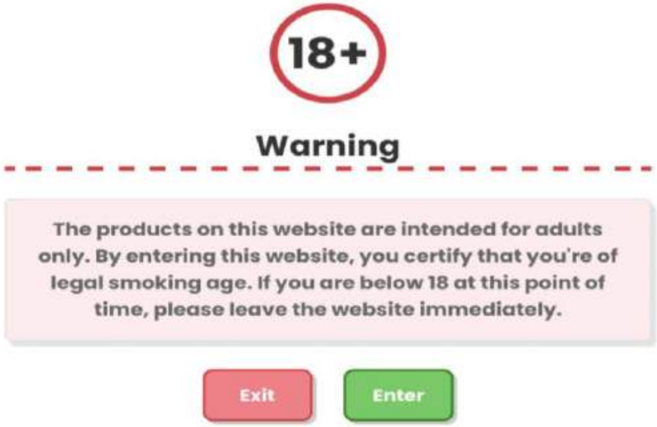

2. **Regulatory language:** These include warnings against minors accessing the website and using the products and nicotine disclaimers.

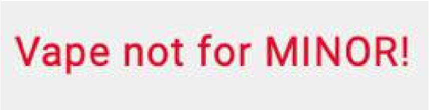

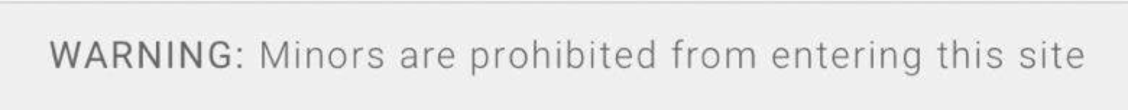

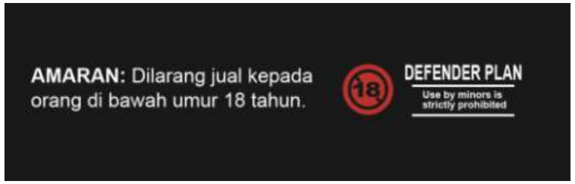

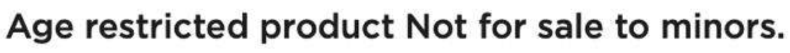

AGE POLICY

This site contains information regarding electronic cigarettes. You must meet the minimum age requirement of your country and state to view any pages of the website and/or purchase electronic cigarettes and electronic cigarette related products from this website.

Falsifying your age for the purpose of purchasing products from this web site is strictly illegal and is punishable by law. Purchasing items on behalf of a minor is prohibited and punishable by law. We reserve the right to ask for identification upon our discretion. By shopping at our store, you are confirming that you are of legal age in your country and state to purchase electronic cigarette products. We reserve the right to deny any order if we think it is placed by a minor. Providing a false declaration under penalties of perjury is a criminal offense.

WARNING: THIS PRODUCT CONTAINS NICOTINE. NICOTINE IS AN ADDICTIVE CHEMICAL

WARNING : THIS PRODUCT CONTAINS NICOTINE. NICOTINE IS AN ADDICTIVE CHEMICAL. ABOVE 18+ ONLY.

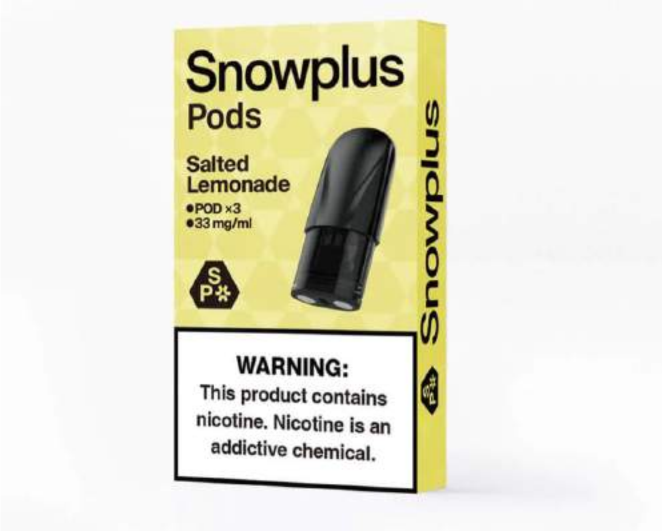

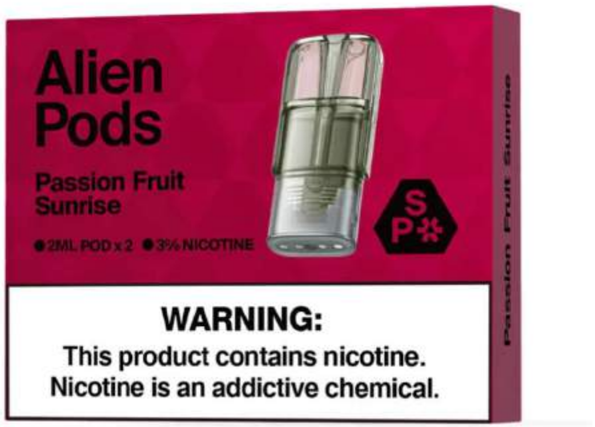

3. **Health-related claims:** These include claims that e-cigarettes are healthy and safe in comparison to conventional cigarettes.

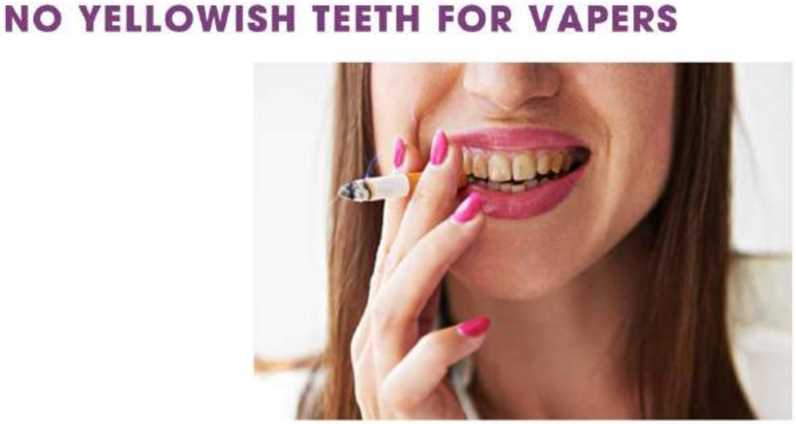

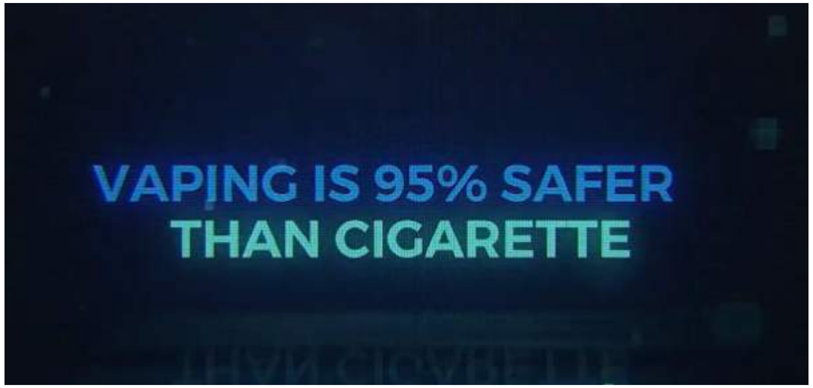

The True Facts About Electronic Cigarettes

Despite misinformation by the media, news or governments, here are the true facts about electronic cigarettes:

Public Health England

Public Health England (PHE), the executive agency of the United Kingdom's Department of Health: "Our new review reinforces the finding that vaping is a fraction of the risk of smoking, at least 95% less harmful, and of negligible risk to bystanders. Yet over half of smokers either falsely believe that vaping is as harmful as smoking or just don't know." – Professor John Newton, Director for Health Improvement, PHE

> *"Nearly half the population (44.8%) don't realise e-cigarettes are much less harmful than smoking."*

> *"There is no evidence so far that e-cigarettes are acting as a route into smoking for children or non-smokers."*

"Smoking remains England's number one killer and the best thing a smoker can do is to quit completely, now and forever. E-cigarettes are not completely risk free but when compared to smoking, but evidence shows they carry just a fraction of the harm. The problem is people increasingly think they are at least as harmful and this may be keeping millions of smokers from quitting. Local stop smoking services should look to support e-cigarette users in their journey to quitting completely." – Professor Kevin Fenton, Director of Health and Wellbeing, Public Health England

Queen Mary University of London, Wolfson Institute of Preventive Medicine: "E-cigarettes are the most important development so far, not only in the treatment of tobacco dependence, but in tobacco control generally. If e-cigarettes are allowed to continue to compete with conventional cigarettes and develop further so that they give more and more smokers what they want from their cigarettes, smoking could virtually disappear." – Professor Peter Hajek, Director of the Wolfson Institute of Preventive Medicine's Tobacco Dependence Research Unit, Queen Mary University of London

"I understand, though do not approve of, the anti-e-cigarette campaigns driven by commercial motives. It is harder to comprehend campaigners motivated by misguided ideology. It is patently obvious that disseminating misleading scare stories about e-cigarettes puts smokers off making the switch to e-cigarettes which would undoubtedly benefit their health. Representatives of some well-known public health and medical bodies in the UK and USA went on record with assertions that e-cigarettes are as harmful as or even more harmful than cigarettes. This kind of deception must be in breach of professional ethics, whatever the motivation behind it."

"My reading of the evidence is that smokers who switch to vaping remove almost all the risks smoking poses to their health."

Cancer Research UK: "E-cigarettes have huge potential to save lives by providing an alternative to smoking. Yet this can only be realized if we address negative harm perceptions and communicate honestly with the public. For those trying with e-cigarettes, this is a positive choice that should be supported." - Linda Bauld, CRUK/BUPA Chair in Behavioural Research for Cancer Prevention at Cancer Research UK, Professor of Health Policy at the University of Stirling, and Deputy Director of the UK Centre for Tobacco and Alcohol Studies

"Fears that e-cigarettes have made smoking seem normal again or even led to people taking up tobacco smoking are not so far being realised based on the evidence assessed by this important independent review. In fact, the overall evidence points to e-cigarettes actually helping people to give up smoking tobacco."

"It would be tragic if thousands of smokers who could quit with the help of an e-cigarette are being put off due to false fears about their safety." – Professor John Newton, Director for Health Improvement, PHE

Is It Safe To Switch To Vaping?

According to Public Health England, e-cigarettes are 95% less harmful to your health than normal cigarettes based on a comprehensive review of the scientific evidence in 2015 and again in 2018.

The Boston University School of Public Health also concluded that the level of carcinogenic dust in electronic cigarettes thousand times lower is than in normal cigarettes.

4. **Smoking cessation-related claims:** These includes claims that e-cigarettes aid in smoking cessation.

Breaking through the traditional cigarette and go with the flow.

We want you to be part of the solution to end combustible smoking. We believe that FLOW is the first step to replace the cigarette. Our mission is to switch the current adult smokers to a smoke-free product where FLOW is a better alternative to continued smoking.

TESTIMONIALS

"I smoked for 26 years - unable to truly quit. I haven't had a cigarette in 4.5 years now because I make the choice to vape.

★★★★★

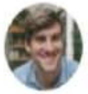

N Cook

"I wasn't a heavy smoker but when I started in that first, and now I was hooked after one year. Now that I've been vaping for a year plus, its...

★★★★★

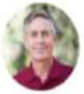

Rene

"If not for vaping I would already be dead. I started smoking at a very early age. 1 pack turned to 2. At the end of my smoking I was a 4...

★★★★★

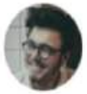

NJ

NanoSTIX is Malaysia's pioneer manufacturer for close-pod electronic cigarettes technology which was founded in January 2017. Headquartered in Malaysia, the company has established the business by selling over 800k units over the past 2 years. NanoSTIX's main goal is to create a world free of cigarette by helping others stop smoking. In terms of design, NanoSTIX device is handy and easy to carry everywhere. Our NanoPOD produces less smoke compared to others. Fantasy Lab, a subsidiary company by NanoSTIX Venture is helping billions of smokers to quit smoking using the first and fastest TRT (Tobacco Replacement Therapy) product that was able to help smokers to stop smoking in less than one month. With the tagline, 'Designed for Future', NanoSTIX practice vibrant and energetic office culture with fun balanced working environment. In 2022, we will launch new product called NanoSTIX Neo, with bigger capacity, better battery lasting and more powerful. Don't miss you chance to grab it once we launch it.

5 Ways to Quit Smoking Slowly for Malaysians

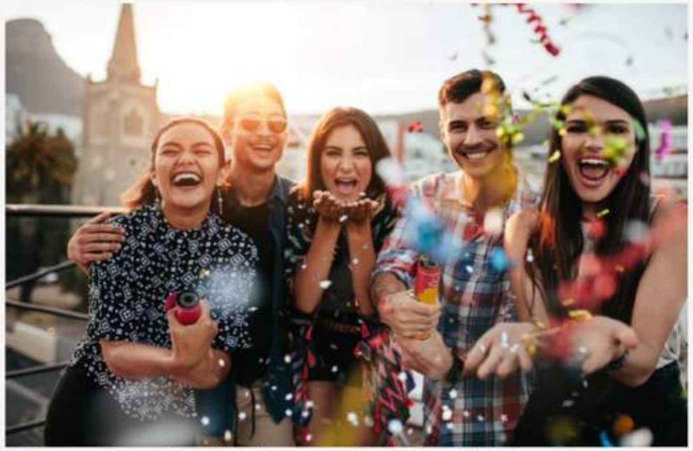

Celebrate your no-smoking journey with your best buddies.

According to the National Health and Morbidity Survery 2015, 52.3% of Malaysians cigarette smokers were looking to quit the habit.[1]. Likewise, 59.5% of smokers thought about quitting after noticing health warning labels on cigarette boxes. For a large number of smokers, their desire to quit the habit is driven by a variety of reasons, such as health, family, or finances. However, quitting is not easy, and relapsing is not uncommon. The good thing, though, is that each shot at quitting is another small step towards the day when you can finally say '*Saya sudah berhenti merokok*'!

Housemates John Ng, 30 and Reuben Michael, 31, agree. They have come to realise that half the battle is having the mental strength and fortitude to resist the cravings and urges to smoke.

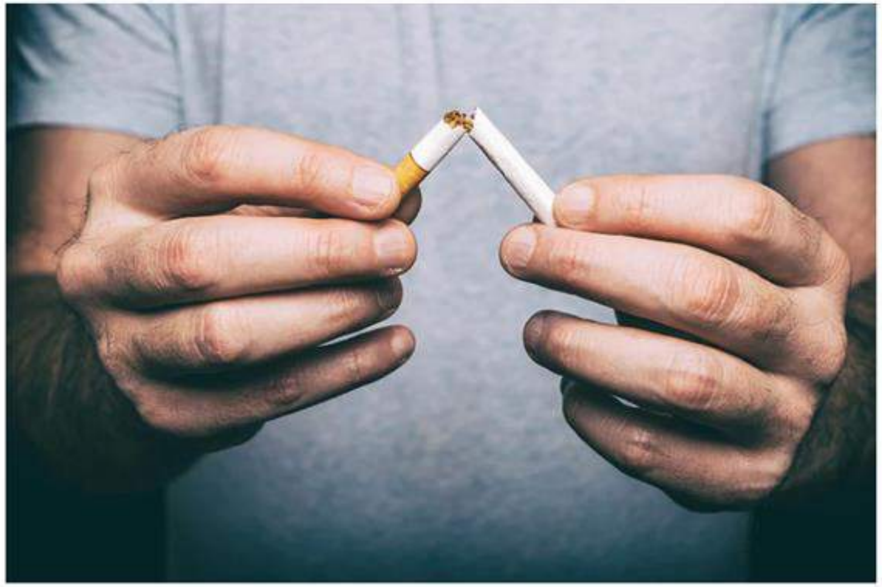

Time to Take Control! 4 Effective Ways to Quit Smoking

Since 2015, Binjai Juice has helped countless smokers to quit smoking by switching to vaping. Our mission is to help you curb your smoking habits and regain your quality of life.

With passion and quality in mind, we want to deliver satisfaction in every puff possible thru our wide range of flavors and the strength selection of our menus.

A suitable alternative might be the thing that helps you quit smoking for good.

Quitting cold turkey has worked for many people, and it may work for you. But if you're worried that it could be too big a ste you can consider an alternative to cigarettes.

Nicotine gum and patches allow you to soothe your craving without having to light up. They're available at the pharmacist, b you should speak to your doctor about them before buying.

You can also consider switching to vape, which can be a suitable replacement for smoking without the burned smell or the messy ash and butts. If you choose this, make sure you go with a trusted brand that offers quality products, like any of the RELX Infinity and Essential lines.

5. **Reduced secondhand smoke/ smoke-free environment claims:** These include claims that vaping produces fewer toxic vapors than cigarettes, reduces exposure to secondhand smoke and creates a smoke-free environment.

Our mission:

To provide a one stop center for all your vaping needs in a holistic lifestyle and SMOKE FREE ambience

What Is The Difference Between Vaping & Smoking?

In general the way how you inhale the smoke of a cigarette differs from the vapor of a box mod. This is because of the composition, amount and thickness of the vapor. When you smoke a cigarette, solid particles will be inhaled because it comes from a burning process. In contrast to vaping with a vaporizer, you will inhale aerosol/vapor.

No Disgusting Smell

Apart from the fact that the smoke from cigarettes inhaled by others has a negative effect on the health, it also smells disgusting. The vapor from vaporizers mostly smells very sweet and doesn't affect the health of others.

No Tar, Carbon Monoxide & More Chemical Dust

In contrast to the 4 (with nicotine 5) ingredients in e-liquid, the contains of cigarettes has way more dangerous substances and chemical dust. Apart from the fact that carbon monoxide is released by a burning process, it also releases tar, benzene, formaldehyde, acetone and a lot more chemical dust we don't even know what is inside.

SECOND HAND SMOKE - HIGH POTENTIAL TO GET CANCER

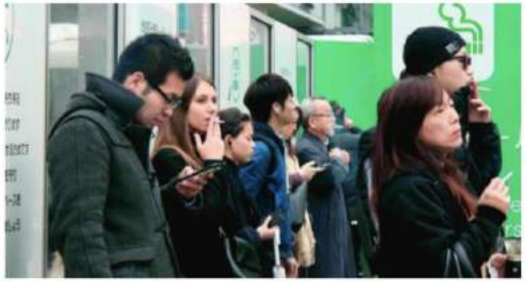

IMAGE BY TSUNAGU JAPAN

Cancer also kills nonsmokers who have a 20 to 30 percent greater chance of developing lung cancer if they are exposed to secondhand smoke at home or work.

An average of130,659 Americans (74,300 men and 56,359 women) died of smoking-attributable lung cancer each year between 2005 and 2010. Exposure to secondhand smoker causes approximately 7,330 lung cancer deaths among nonsmokers every year.

This brief exposure probably isn't a huge deal, but second hand vape aerosol (the "smoke" from vaping) is definitely a thing, even if it smells like candy. It's not clear how harmful secondhand vaping is since vaping is still relatively new. Its long-term effects are still being investigated.

Public Health England

Public Health England (PHE), the executive agency of the United Kingdom's Department of Health: "Our new review reinforces the finding that vaping is a fraction of the risk of smoking, at least 95% less harmful, and of negligible risk to bystanders. Yet over half of smokers either falsely believe that vaping is as harmful as smoking or just don't know." – Professor John Newton, Director for Health Improvement, PHE

"Nearly half the population (44.8%) don't realise e-cigarettes are much less harmful than smoking."

6. **Ability to smoke anywhere and convenience of use:** This promotional messaging that e-cigarettes can be carried and used anywhere with convenience without any mess and need to dispose.

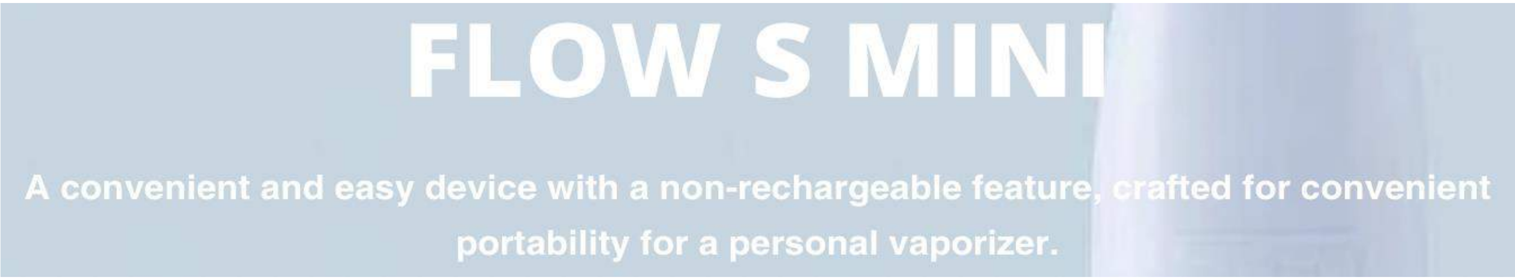

The Uwell Caliburn A2S Pod Kit is a pocket-friendly pod system for vaping on the go. Integrated a 520mAh rechargeable battery into the lightweight aluminum alloy chassis, the Caliburn A2S adopts a draw activated firing system with constant voltage output reaching up to 15W. Featuring a silicone stoppered side filling system to hold 2.0ml of your favorite juices , the Caliburn A2S Pod comes with an integrated 1.2ohm mesh coil to provide delicious and smooth vapor. Moreover, the Caliburn A2S is also compatible with the 0.9ohm Caliburn A2 Pod which features a top filling design.

FEATURES : **RECHARGABLE** TYPE C CABLE – **no more worries of battery running out before juice**

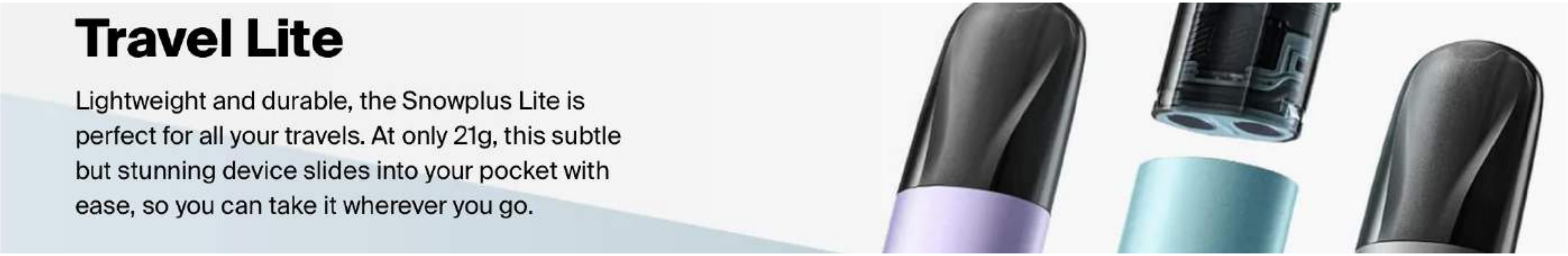

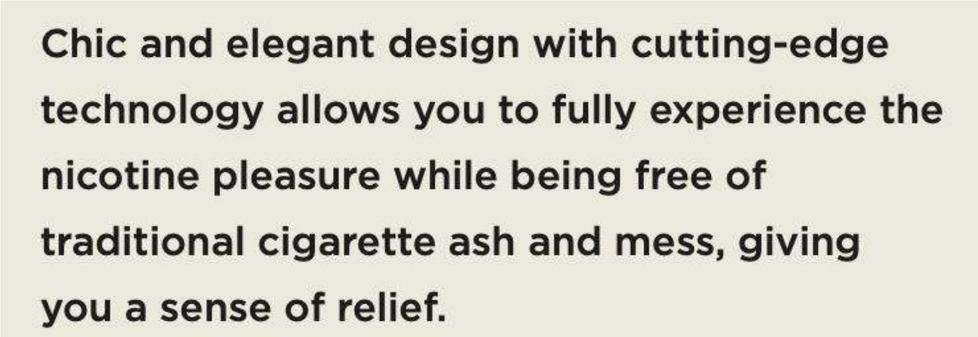

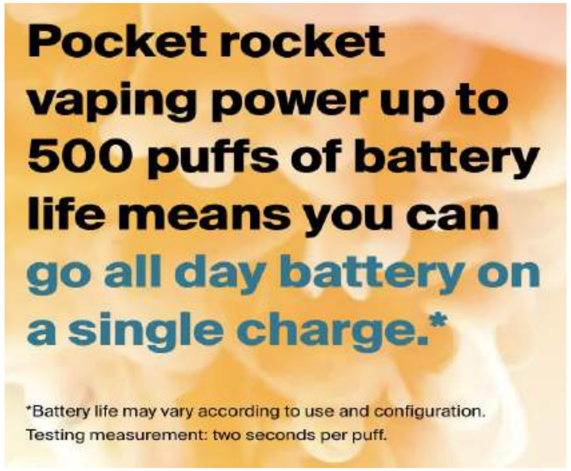

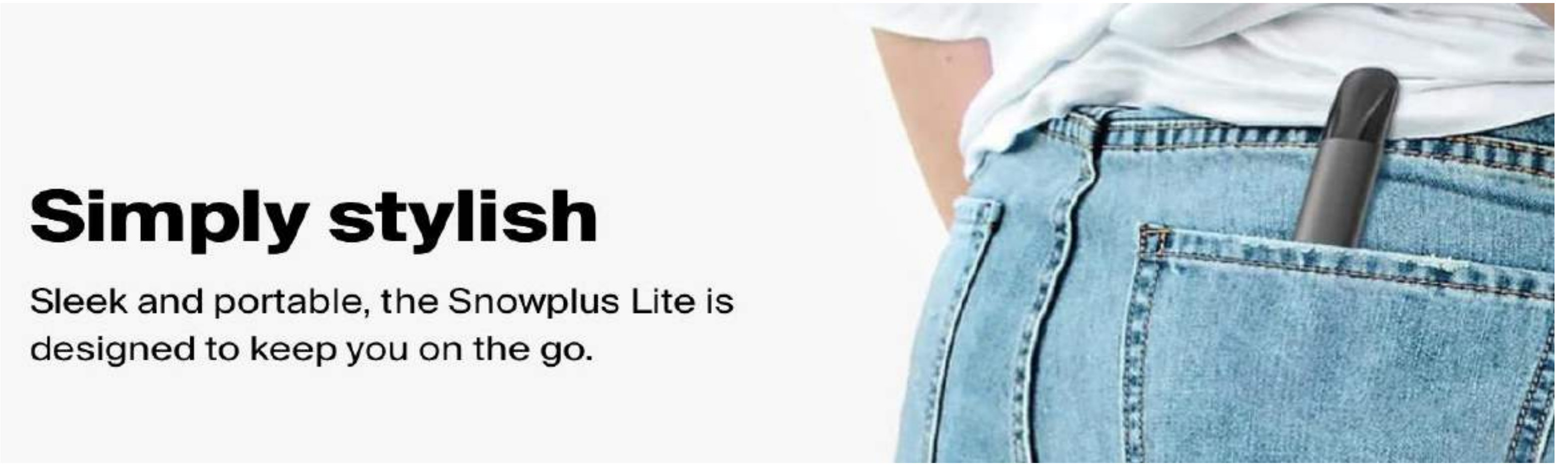

7. **Cheaper:** This includes claims that e-cigarettes are more cost effective that conventional cigarettes and help save money in the long run.

Switching to vaping can save you up to 92% if you smoke a pack a day. However, there are cases when vaping can cost more than smoking.

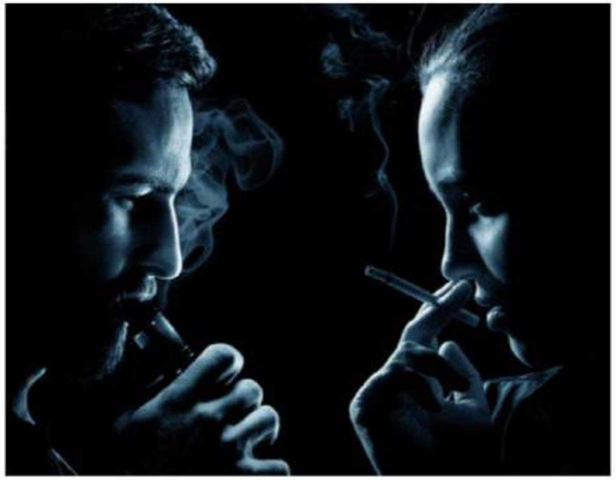

How Much Does an E-Cigarette Cost?

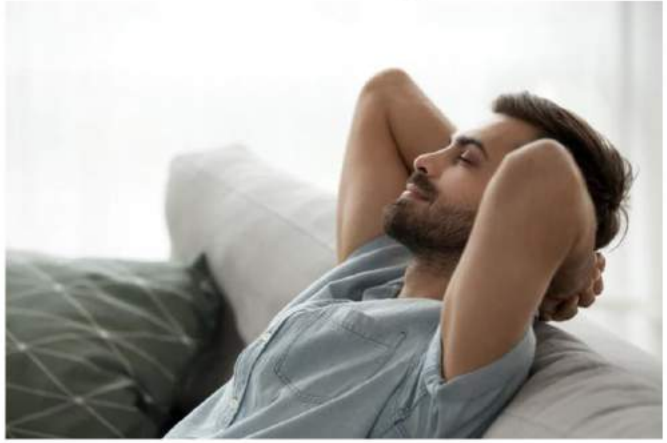

You could finally free yourself from cigarette expense after switching to e-cigarette.

You Will Save Ringgit in the Long Run

Switching to vape can also help you to save some ringgit, opening you up to other, more adventurous possibilities. For instance, the money you save can be used for activities like travelling to holiday destinations or enjoying some good food with friends.

Is It True That Vaping Is More Cost-Effective Compared To Smoking?

Generally vaping is much more cost effective compared to smoking normal cigarettes.

Premium Cigarettes : RM17.50/pack/day | RM525/month
Tax free/Smuggle Cigarettes : RM7/pack/day | RM210/month
Binjai Juice : RM25/week | Rm100/month

8. **Modern:** Claims of modernity include promotional messages of e-cigarettes as a revolutionary way of smoking, and advertising of the device's advanced technology and design.

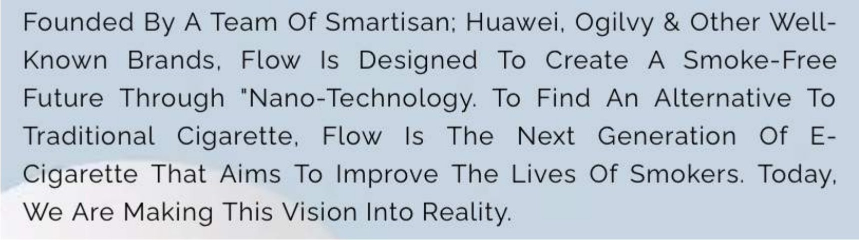

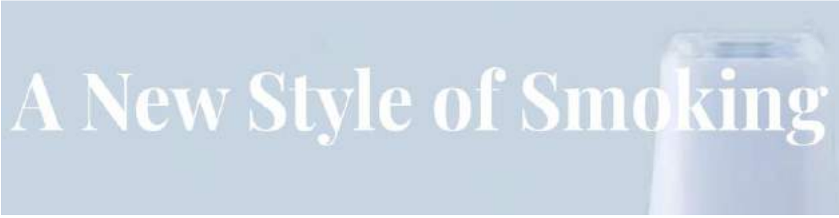

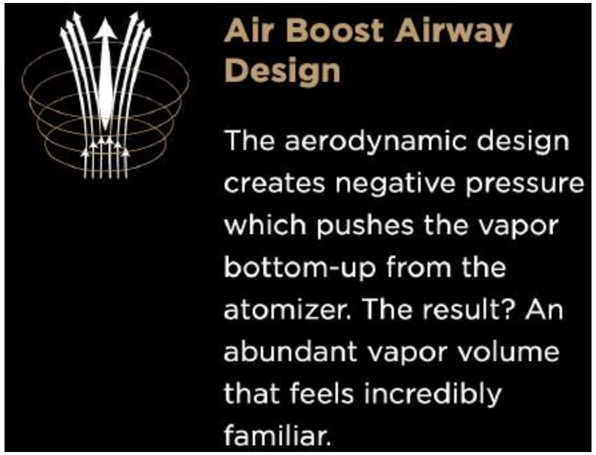

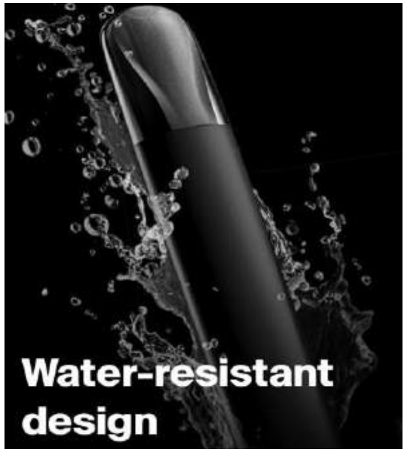

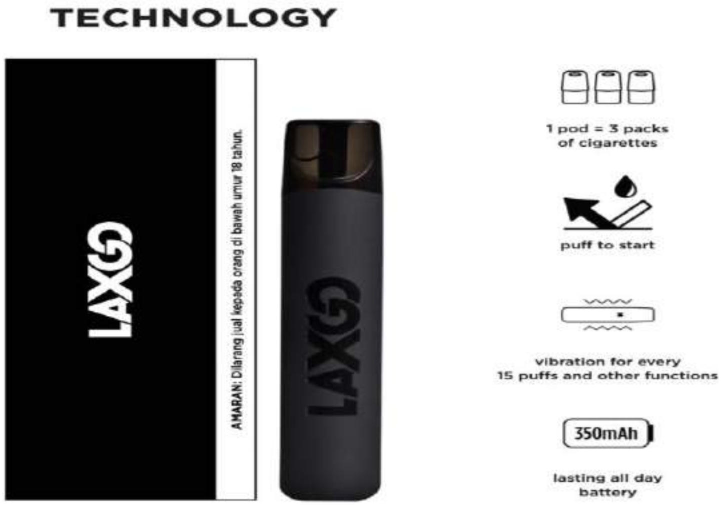

9. **Social enhancement claims:** These include claims that vaping increases social status, popularity, and sexual appeal of the vaper.

Millennials are switching to vape after discovering its many benefits.

Vaping is becoming increasingly popular amongst people of all ages. In fact, a growing number of millennials are hastening to make the switch to vape for FOMO (fear of missing out). In the same way, a significant number of people, including millennials, have chosen to switch after discovering the numerous benefits of vaping. For instance, they need no longer worry about smelling like an ashtray and may even find their chances of getting a date increasing.

Wake Up Your Social Life! The Benefits of Vaping Over Smoking Cigarettes

58% of People Dislike the Idea of Dating Smokers, and That's a Fact!

Finding the perfect present for your smoker boyfriend is always tricky. On the one hand, a gift is meant to bring joy, but on the other hand, this might just be the perfect time to hint that his smoking habits are getting you down. Why not buy a special gift for boyfriend that carries a hidden message and helps inspire him to switch to alternatives and inspire them to have a super smooth year?

10. Promotional offers: Discounted prices, giveaway contests, rewards for referrals, and free samples with purchase are some of the monetary incentives used for advertising and promoting e-cigarettes.

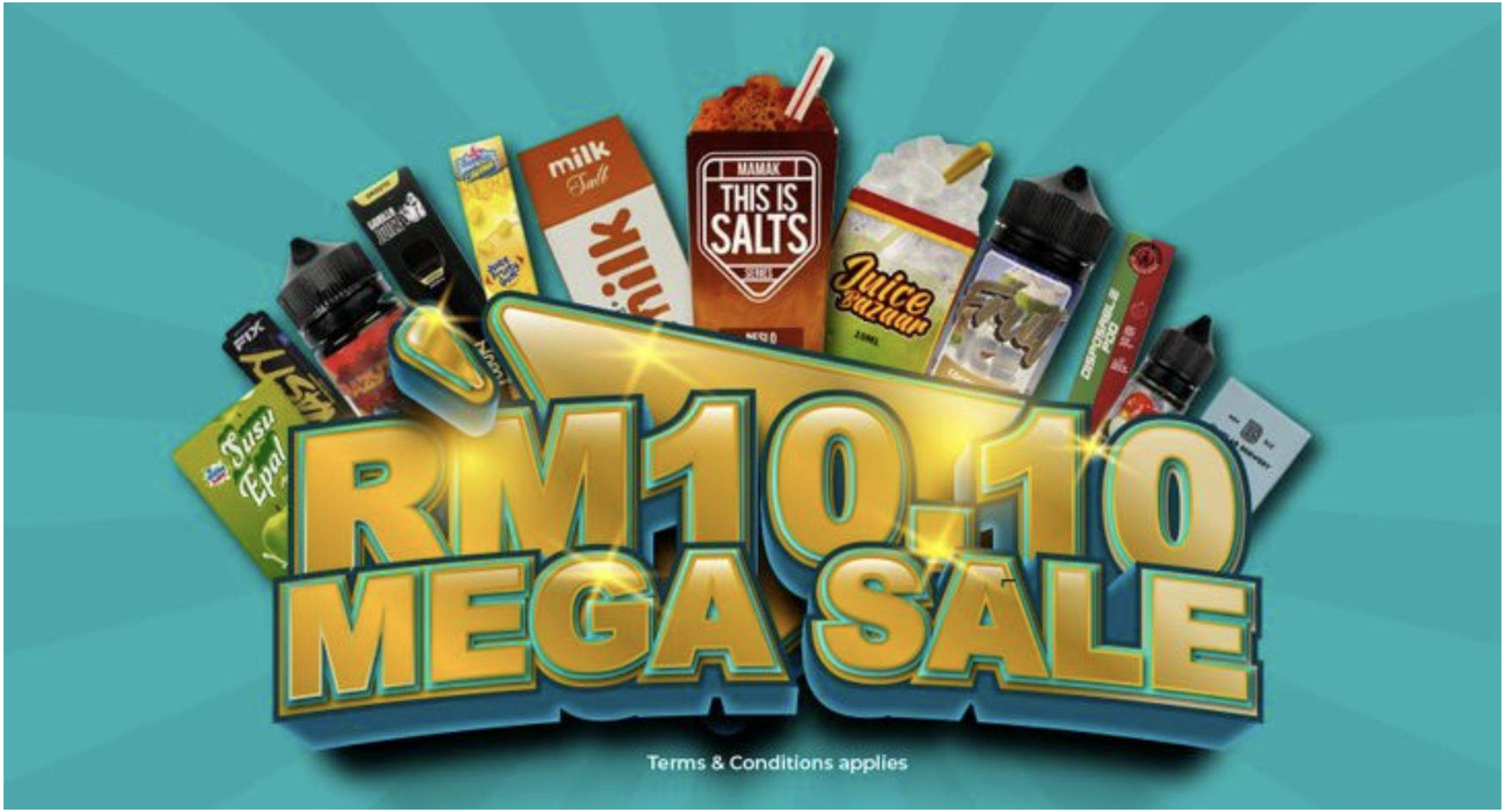

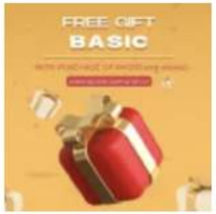

Free Gift - Basic

Purchase a minimum of **RM250.00** and get a free basic gift with your order. Only while stocks last.

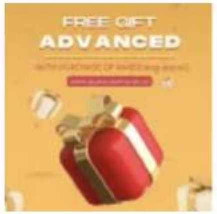

Free Gift - Advanced

Purchase a minimum of **RM450.00** and get a free advanced gift with your order. Only while stocks last.

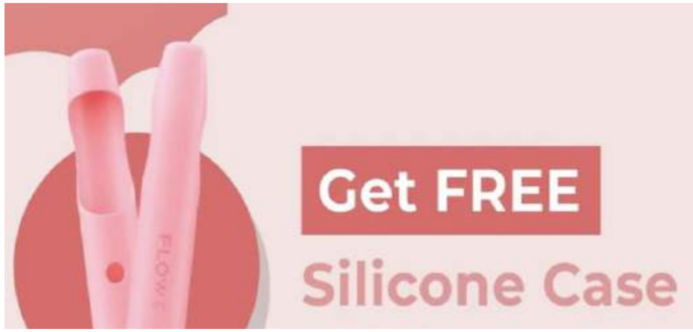

Referrals

Give your friends a reward and claim your own when they make a purchase.

They get
RM15 off coupon

You get
RM15 off coupon

11. Ingredient disclosure: This includes the display of the ingredients present in e-cigarettes and in the composition of e-liquids.

We use the same ingredients common in other e-liquids. These include vegetable glycerin (VG), propylene glycol (PG), food-grade flavorings, and medical-grade nicotine salts for nicotine-containing pods. Menthol is also used for some flavors. You can check the detailed ingredients of each flavor by looking at the back of the packaging.

E-liquid ingredients: propylene glycol, vegetable glycerin, flavourings, nicotine (except zero-nic variants)

What is in e-liquid and are the ingredients harmful to your health?

While there are several thousands of e-liquid brands and flavors available around the world, the fundamental ingredients used to manufacture them are similar, albeit with slight variations within the blend. E-liquid is typically made of:

1. Propylene Glycol (PG)
2. Vegetable Glycerin/Glycerol (VG)
3. Liquid Nicotine
4. Flavoring (Food-Grade)

E-Liquids consist of four major components: Propylene Glycol (PG), Vegetable Glycerin (VG), food-grade food flavourings. For each of the four components, Binjai Juice uses only the highest quality ingredients to create a premium e-liquid with the highest aim for you to #experiencebetter with Binjai Juice.

12. Nicotine promotion: This includes messaging that promotes the use of nicotine or any information related to nicotine.

The nicotine strength in e-liquid is almost always measured in mg/mL (weight of nicotine in mg per ML of e-liquid), where the "per mL" is regularly dropped in conversation or informal writing. Some manufacturers on the other hand use a different measurement in percentage (%), where for example: a 12 mg/mL e-liquid would have '1.2%' nicotine content. Some common nicotine strengths in e-liquid include:

I. 0 mg/mL or 0% (nicotine-free or containing no nicotine)
II. 3 mg/mL or 0.3% (commonly regarded as 'extra light')
III. 6 mg/mL or 0.6% (commonly regarded as 'light')
IV. 12 mg/mL or 1.2% (commonly regarded as 'medium')
V. 18 mg/mL or 1.8% (commonly regarded as 'strong')
VI. 24 mg/mL or 2.4% (commonly regarded as 'extra strong')

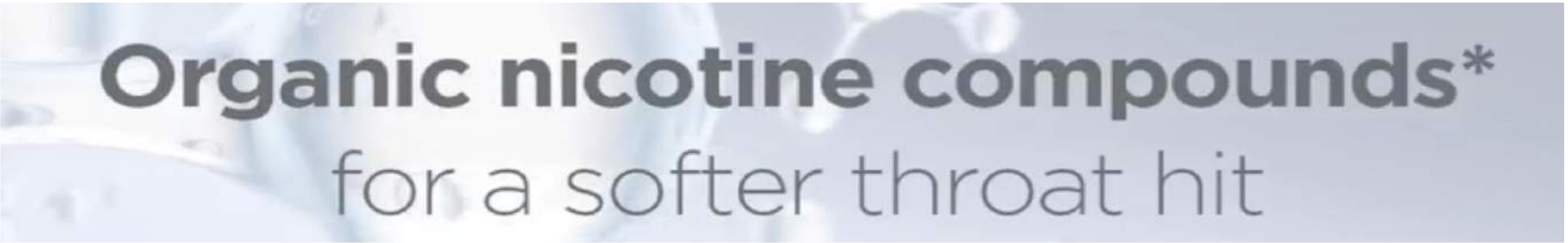
Organic nicotine compounds*
for a softer throat hit

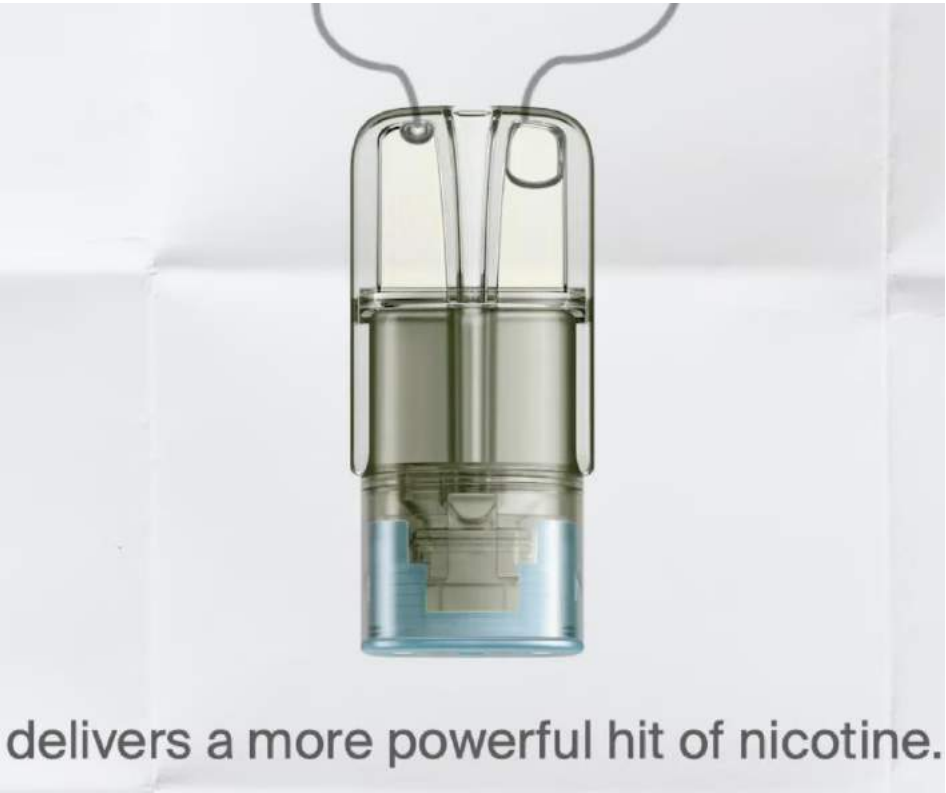
delivers a more powerful hit of nicotine.

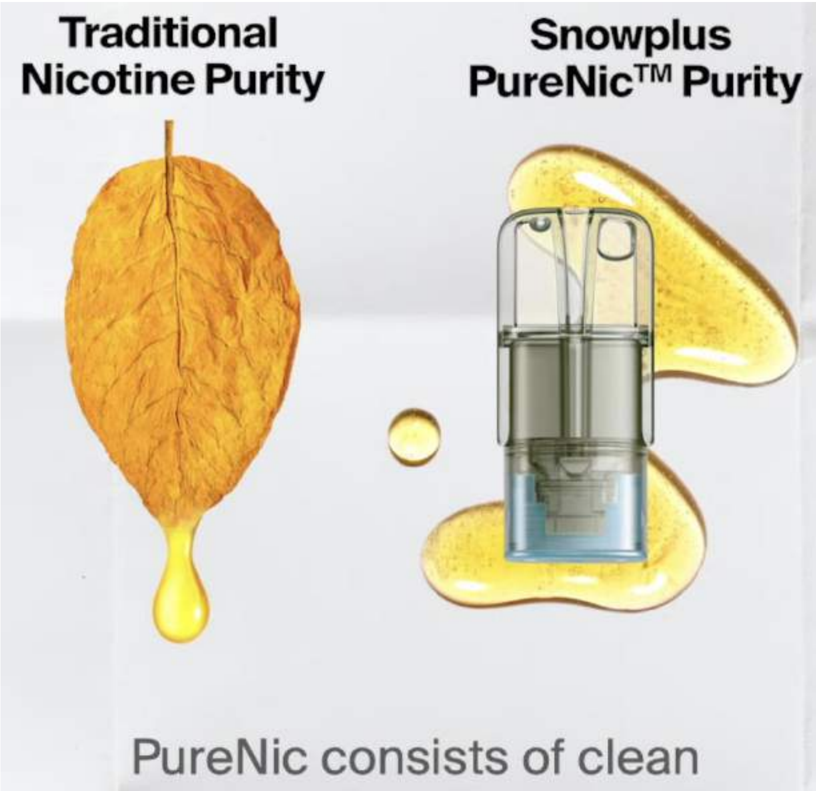
Traditional Nicotine Purity
Snowplus PureNic™ Purity
PureNic consists of clean

Higher dosage of nicotine making it perfect for pod open system device and for mouth to lung vaping. Nasty Salt should never be used in sub-ohm tanks or any dripper styled vape system.

Salt Nicotine Edition

30ml

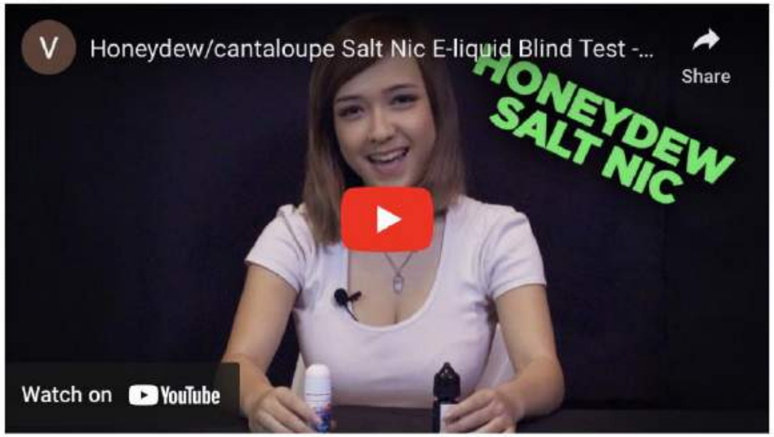

Getting the nicotine hit

But what about the nicotine? Will that still be absorbed through the lungs? No, not really. E-cigarettes deliver nicotine in a different way to tobacco smoke, and most of it gets absorbed through the mouth. This means it's slower to act, which is something a lot of switchers struggle with at first. The solution is to vape at a steadier pace – instead of taking a dozen puffs once an hour, take one or two more often.

If you're a cigar or pipe smoker, however, the fact the nicotine gets absorbed in the mouth is good news. This means you can get the same effect as you did from smoking and *not* inhaling.

Smooth and Satisfying Smoke

Bringing you a powerful refreshing experience and higher absorption rate with Nicotine Salt.

ECO NIC

Eco Nic (Eco Nicotine) aims in giving better taste and better satisfaction.

The New Generation of Nicotine Salt

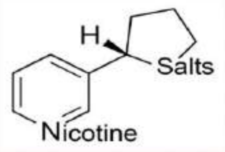

Faster absorption for a stronger hit
Taste and feel like a real cigarette

Differences	Freebase Nicotine	Nicotine Salts
Throat Hit	Strong	Smooth
Nicotine Strength	Low	High
Head Buzz	Slow	Fast
Ideal Wattage	Low & High	Low
Flavours	Complex	Simple

Head Buzz

Nicotine salts will do the job just right. With the high concentration of nicotine and the fast absorption into your bloodstream, the head buzz you'll get feels very similar to having your first puff of cigarette in the morning.

Freebase nicotine is much more delicate. It doesn't hit you as hard but because of it, you're able to vape more frequently and enjoy the flavours much more.

13. **Nicotine content disclosure:** The open disclosure of amount of nicotine present in vape products displayed on the websites, and levels of nicotine for customers to choose from.

NICOTINE LEVEL 30mg

-2% (20mg) nicotine strength

- Nicotine Strength: 2%

PUFF : UP TO 1200 PUFFS

NIC STRENGTH : 5%

7 DAZE US SALTNIC 30ML

Synthetic Nicotine

HTPC

0 mg

03 mg

06 mg

12 mg

15 mg

18 mg

22 mg

25 mg

30 mg

35 mg

40 mg

50 mg

FILTER BY NICOTINE

35mg (19)

35mg + plain discrete cosmetic bottle (19)

50mg (19)

50mg + plain discrete cosmetic bottle (19)

KARDINAL HTPC 30ML Specification

- Volume : 30ML
- MG : 12mg , 18mg , 22mg
- Nicotine : 1.2%

14. Instructions to use: These include instructions on how to use the device, vaping guides for beginners and instructions on how to choose the right type and strength of nicotine.

How does a pod work?

▶ Play video

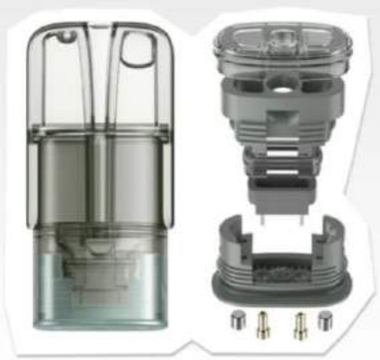

Your device automatically switches on each time you pull in air through the mouthpiece of your vape, prompting the atomizer to heat the e-liquid and transform it into vapor.

WHAT LEVEL OF NICOTINE SALT SHOULD I TRY?

For guidance:

- If you vape 0mg freebase liquids, there is no point to try nicotine salts. Nicotine salts are meant to deliver a strong nicotine hit.
- If you vape 3mg/6mg freebase liquids, you should be able to handle 25mg/30mg nicotine salts.
- If you vape 12mg's or higher, you're in the middle ground. You could go for 25mg/30mg for less of a nicotine buzz or prefer 50mg nicotine salts for a stronger buzz.
- If you are a smoker looking to switch to vape - than 50mg is definitely the way to go. You will get your nicotine fix quickly after only a few puffs which will help you get off smoking for good.

Home > Vape Beginner's Guide > How to Vape for the First Time: Vaping Dos & Don'ts for Beginners

How to Vape for the First Time: Vaping Dos & Don'ts for Beginners

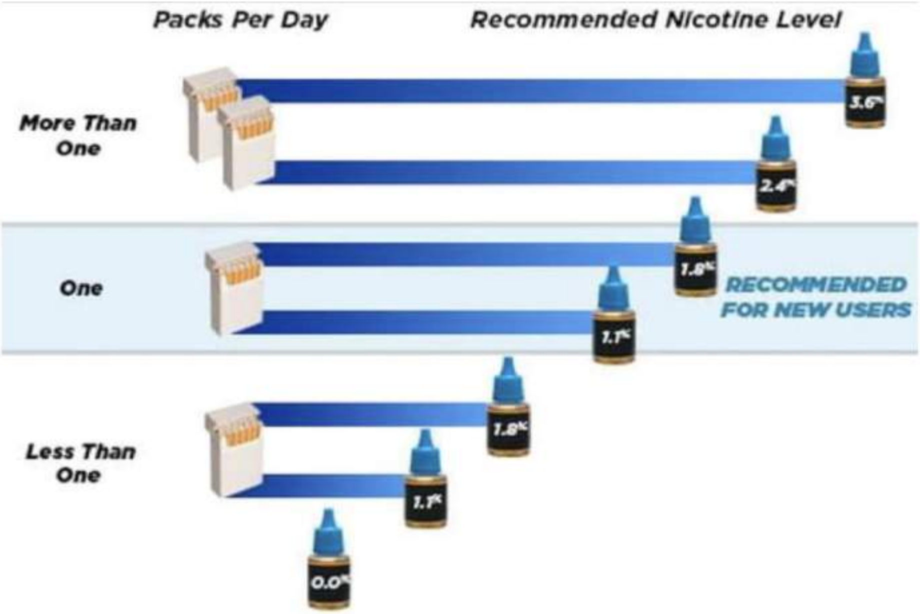

Which should I choose?

Get Freebase Nicotine Juices If :	Get Nicotine Salt Juices If :
• You're a social vaper/smoker<br>• You want to vape complex flavours<br>• You want to have lots of vape clouds<br>• You're vaping/smoking out of habit	• You want to quit smoking<br>• You've tried vaping before and it didn't work<br>• You want a small, compact device<br>• You want a quick fix for your nicotine cravings

How do you know the nicotine content that you need?

One of the mistakes you should not make when deciding on the nicotine content of your e-liquid is to refer to overly simplistic calculations such as: I smoke this number of cigarettes per day, so I need an e-liquid containing this much nicotine.

In fact, as the Professor points out, this is not the right method to use for a number of reasons:

- The nicotine content in a cigarette varies according to its brand and model. It may be from 6 to 17 mg, but smokers only absorb 1 to 2 mg.
- The nicotine content specified by manufacturers on packs is based on smoking machines which do not reproduce a human's tobacco consumption. These contents are generally underestimated.
- The link between cigarettes/day and nicotine absorption is not linear, but more like an inverted U-shaped curve.

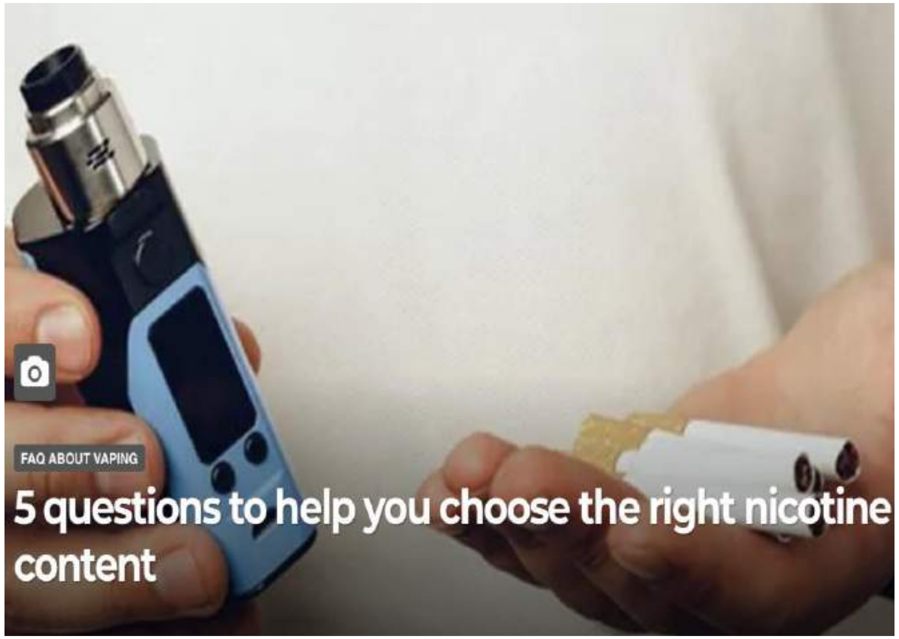

This questionnaire has been the subject of a scientific article (available to access here), so these are not just some questions taken at random. Answering these questions will give you a realistic estimation of your exposure to nicotine, allowing you to infer the dose that you really need.

Questionnaire

Question No. 1

How many cigarettes do you smoke per day, on average?

- Zero = 0 points
- 1 to 5 = 1 point
- 6 to 10 = 2 points
- 11 to 15 = 3 points
- 16 to 20 = 4 points
- 21 and over = 5 points

Question No. 2

After you wake up, how much time passes before you have your first cigarette of the day?

- From 0 to 5 minutes = 5 points
- From 6 to 15 minutes = 4 points
- From 16 to 30 minutes = 3 points
- From 30 minutes to 1 hour = 2 points
- Over one hour afterwards = 1 point

Question No. 3

Indicate, on a scale between 1 and 100, the intensity of your smoking.

- 0 = 0 points
- From 1 to 20 = 1 point
- From 21 to 40 = 2 points
- From 41 to 60 = 3 points
- From 61 to 80 = 4 points
- From 81 to 100 = 5 points

Question No. 4

Indicate the total quantity of smoke that you inhale every day. 10 means that you inhale so much that you could not inhale more smoke, even if you tried.

- 0 = 0 points
- 1 or 2 = 1 point
- 3 or 4 = 2 points
- 5 or 6 = 3 points
- 7 or 8 = 4 points
- 9 or 10 = 5 points

Question No. 5

Finally, indicate the quantity of nicotine contained in your cigarettes written on their pack.

- From 0.1 to 0.5 mg = 1 point
- From 0.6 to 0.7 mg = 2 points
- 0.8 mg = 3 points
- 0.9 mg = 4 points
- 1 to 1.3 mg = 5 points

Results

Now add up the number of points that you scored for each question. With a minimum of 0 and a maximum of 25.

- Between 0 and 4 points: you absorb between 0 and 7 mg of nicotine per day (average of 2.4 mg/day).
- Between 5 and 9 points: you absorb between 7 and 14 mg of nicotine per day (average of 10 mg/day).
- Between 10 and 14 points: you absorb between 14 and 21 mg of nicotine per day (average of 17 mg/day).
- Between 15 and 19 points: you absorb between 21 and 28 mg of nicotine per day (average of 24 mg/day).
- Between 20 and 25 points: you absorb over 30 mg of nicotine per day.

15. **Slogans:** These are catchphrases exclusive to e-cigarette manufacturers or retailer brands used as an advertising strategy.

Stop Smoking & Let's change to Vape.

Our vision:
Stop Smoking and Start Vaping

EAT, SLEEP, VAPE, REPEAT

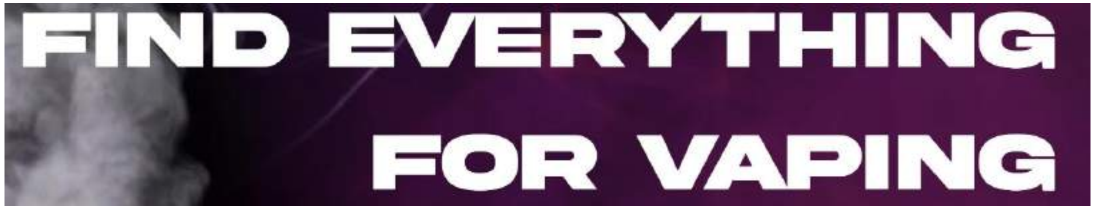

Royale Vape
EST 2015
Vape Like Royalty

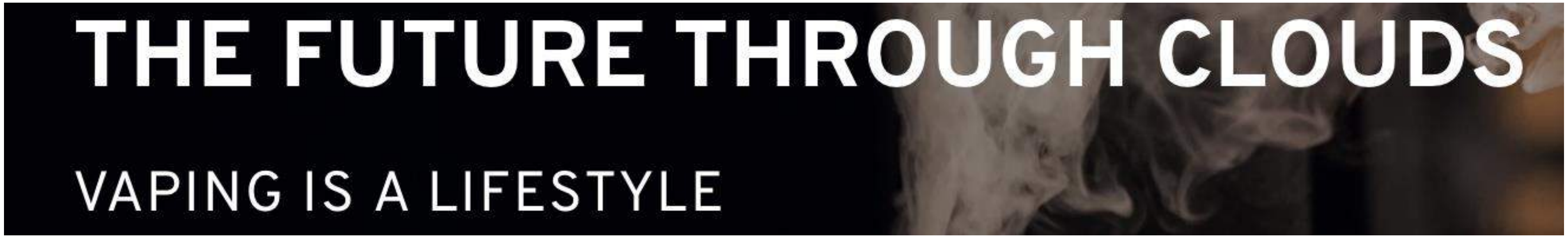

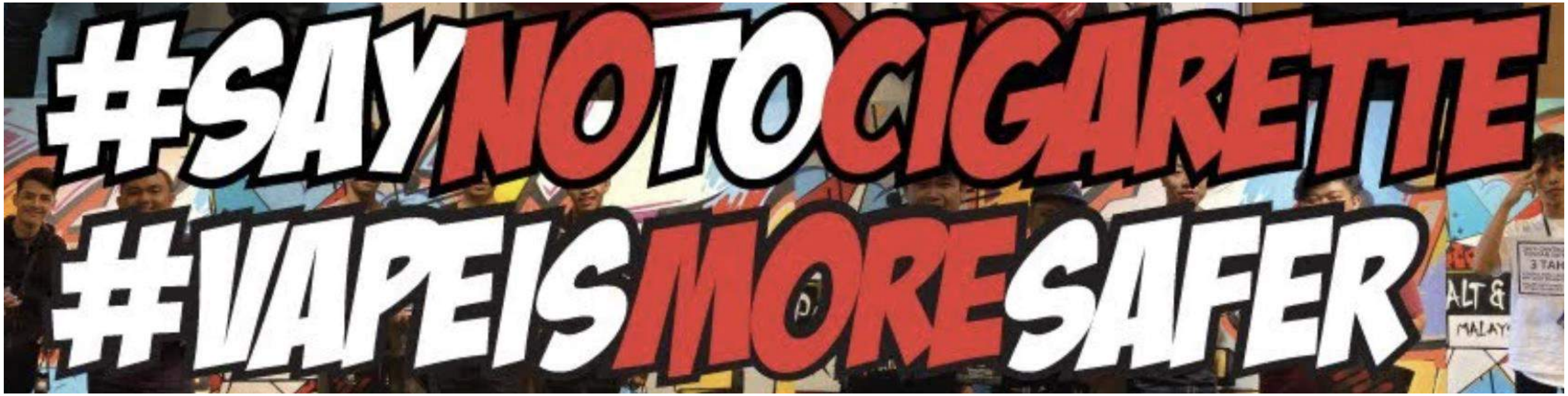

16. Product diversity: The different types on products found on internet e-cigarette vendor websites include starter kits, rechargeable vapes, disposable vapes, and e-liquids. Products are classified as nicotine and non-nicotine vape products.

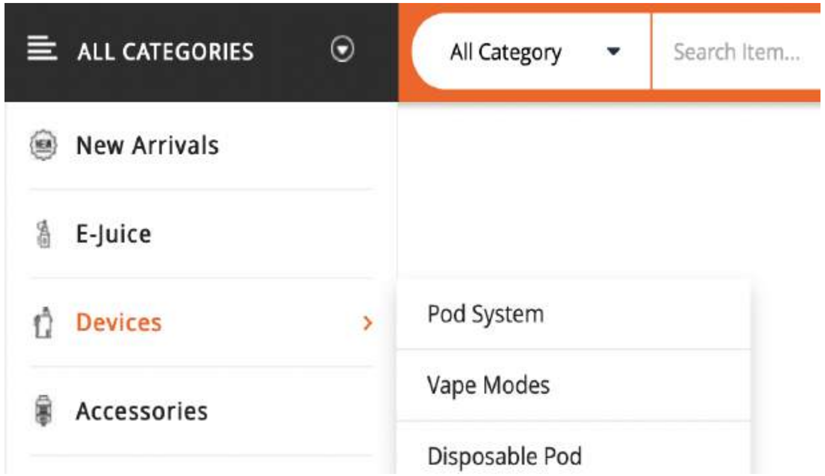

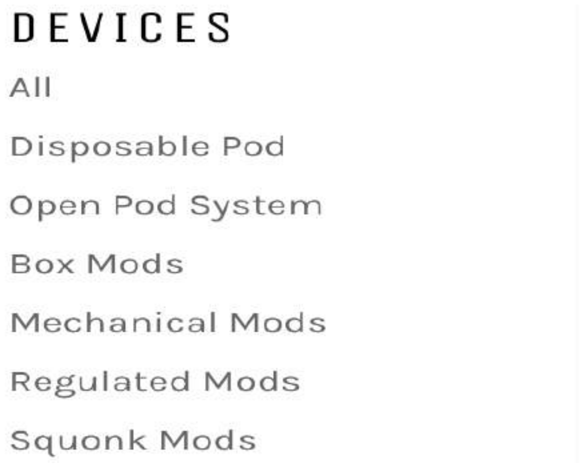

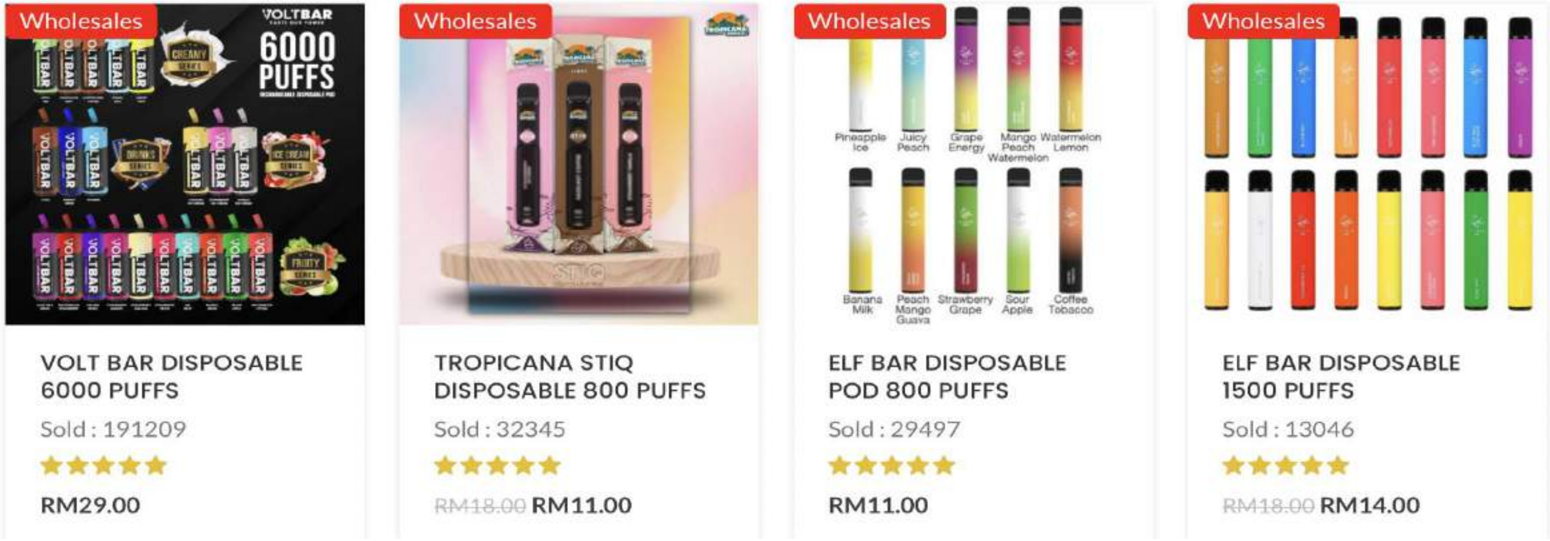

Supplement: Supplementary file 1 — Supplementary Material 1 [file 13011_2024_592_MOESM1_ESM.pdf]
